# Efficient nitrite determination by electrochemical approach in liquid phase with ultrasonically prepared gold-nanoparticle-conjugated conducting polymer nanocomposites

**DOI:** 10.3389/fchem.2024.1358353

**Published:** 2024-08-06

**Authors:** M. Faisal, M. M. Alam, Jahir Ahmed, Abdullah M. Asiri, Jari S. Algethami, Raed H. Altholami, Farid A. Harraz, Mohammed M. Rahman

**Affiliations:** ^1^ Promising Centre for Sensors and Electronic Devices (PCSED), Advanced Materials and Nano-Research Centre, Najran University, Najran, Saudi Arabia; ^2^ Department of Chemistry, Faculty of Science and Arts, Najran University, Najran, Saudi Arabia; ^3^ Department of Chemical Engineering, Faculty of Engineering and Technology, Z. H. Sikder University of Science and Technology (ZHSUST), Shariatpur, Bangladesh; ^4^ Center of Excellence for Advanced Materials Research (CEAMR), King Abdulaziz University, Jeddah, Saudi Arabia; ^5^ Department of Chemistry, Faculty of Science, King Abdulaziz University, Jeddah, Saudi Arabia; ^6^ Department of Chemistry, College of Art and Science, Prince Sattam bin Abdulaziz University, Wadi Al Dawasir, Saudi Arabia; ^7^ Department of Chemistry, Faculty of Science and Arts at Sharurah, Najran University, Sharurah, Saudi Arabia

**Keywords:** Au-NPs/PPyC/SrTiO3 nanocomposites, nitrite detection, glassy carbon electrode, differential pulse voltammetry, environmental remediation

## Abstract

An electrochemical nitrite sensor probe is introduced herein using a modified flat glassy carbon electrode (GCE) and SrTiO_3_ material doped with spherical-shaped gold nanoparticles (Au-NPs) and polypyrrole carbon (PPyC) at a pH of 7.0 in a phosphate buffer solution. The nanocomposites (NCs) containing Au-NPs, PPyC, and SrTiO_3_ were synthesized by ultrasonication, and their properties were thoroughly characterized through structural, elemental, optical, and morphological analyses with various conventional spectroscopic methods, such as field-emission scanning electron microscopy, energy-dispersive X-ray spectroscopy, high-resolution transmission electron microscopy, powder X-ray diffraction, X-ray photoelectron spectroscopy, and Brunauer–Emmett–Teller method. The peak currents due to nitrite oxidation were characterized in detail and analyzed using conventional cyclic voltammetry (CV) as well as differential pulse voltammetry (DPV) under ambient conditions. The sensor response increased significantly from 0.15 to 1.5 mM of nitrite ions, and the sensor was fabricated by coating a conducting agent (PEDOT:PSS) on the GCE to obtain the Au-NPs/PPyC/SrTiO_3_ NCs/PEDOT:PSS/GCE probe. The sensor’s sensitivity was determined as 0.5 μA/μM∙cm^2^ from the ratio of the slope of the linear detection range by considering the active surface area (0.0316 cm^2^) of the flat GCE. In addition, the limit of detection was determined as 20.00 ± 1.00 µM, which was found to be satisfactory. The sensor’s stability, pH optimization, and reliability were also evaluated in these analyses. Overall, the sensor results were found to be satisfactory. Real environmental samples were then analyzed to evaluate the sensor’s reliability through DPV, and the results showed that the proposed novel electrochemical sensor holds great promise for mitigating water contamination in the real samples with the lab-made Au-NPs/PPyC/SrTiO_3_ NC. Thus, this study provides valuable insights for improving sensors for broad environmental monitoring applications using the electrochemical approach.

## Introduction

Nitrates (NO_3_^−^) and nitrites (NO_2_^−^) are chemical compounds that are generally used as food preservatives in processed meats, such as bacon, ham, sausages, and hot dogs. They inhibit the growth of bacteria, particularly *Clostridium botulinum*, which is responsible for causing botulism. These nitrates and nitrites also contribute to the characteristic flavor, color, and aroma of cured and processed meats (Kalaycıoğlu and Erim, 2019; Karwowska and Kononiuk, 2020); moreover, they have been known for their potential to form nitrosamines in the stomach, particularly amines and amino acids. Nitrosamines are considered as carcinogens that cause various cancers, including stomach, esophageal, colorectal, and pancreatic cancers (Bartsch et al., 1989; Nawrocki and Andrzejewski, 2011). Nitrates and nitrites can also be converted into nitric oxide (NO) in the human body through various biological processes; nitric oxide plays a positive role in immune function and neurotransmission. Additionally, nitrates and nitrites are naturally present in foods, such as vegetables, fruits, grains, and water, and these dietary nitrates are generally considered safe for health benefits, such as cardiovascular health support and reduced risk of certain diseases (Bahadoran et al., 2016; Bedale et al., 2016; Huang et al., 2022). However, nitrates can leach into the groundwater and surface water when excess nitrogen-based fertilizers are used in agriculture or when sewage and animal wastes are improperly managed. Elevated nitrate levels in drinking water can pose health risks, particularly for infants and young children, as excessive nitrate consumption can lead to a condition known as methemoglobinemia (or blue baby syndrome), which interferes with the ability of the blood to carry oxygen (Tamaki et al., 2003; Meng et al., 2009; Lu et al., 2023). Thus, to develop a system to monitor nitrite contamination in the environment and concentrations in bodily fluids (blood and urine), a reliable sensor is necessary.

Accordingly, procedures involving chromatography (Hamilton and Lewis, 2006), Fourier-transform infrared (FT-IR) spectrometry (Griffiths and De-Haseth, 2007), laser absorption spectroscopy (LAS) (Edwards et al., 2003), and optical analyses (Hassan et al., 2022; Kang et al., 2023) have been implemented to measure nitrite levels in real samples. However, these techniques are unreliable owing to their poor sensitivities, narrow detection ranges, longer analysis times, higher costs, and lower portabilities. To overcome these problems, researchers are working hard to develop alternative techniques. As a result, voltammetric techniques like differential pulse voltammetry (DPV) and cyclic voltammetry (CV) have become popular detection approaches. In recent years, some reports have claimed that working electrodes made of graphite, glassy carbon, carbon paste, and other noble metals could be successfully applied to detect unknown concentrations of nitrites using voltammetric electrochemical approaches (Kaminskay et al., 2004). However, pure-metal electrodes are limited by poor electron transport and selective electrode surfaces. Alternatively, as reported elsewhere, the functioning electrode surfaces are modified with sensing substrates to develop electrochemical sensors (Terbouche et al., 2016; Tohidinia et al., 2018; Amini et al., 2021; Wang C. et al., 2024).

Amperometry and DPV are commonly used electrochemical methods for detecting trace amounts of toxic contaminants. In this study, the general and reliable voltammetric method was used to develop a nitrite electrochemical sensor. Accurate nitrite detection in aqueous systems, metal composites, metal oxides, and organometallic substances like zinc oxide nanoflowers and reduced graphene oxide (RGO), tin dioxide nanoparticle/RGO hybrids, graphene/polymer nanofibers, and nanocomposites of conducting polymers are well documented. Hence, the present study aimed to develop an electrochemical sensor probe using conductive PEDOT: PSS polymer composited with a sensing layer via metals and metal oxides for reliable electrochemical determination of nitrites to detect trace levels of the target analytes; this would also ensure environmental and health safety in a broad scale. Gold nanoparticles (Au-NPs) and SrTiO_3_ have gained significant attention in various fields owing to their unique optical, electronic, and catalytic properties. In nitrite sensing, Au-NPs are utilized for their ability to detect nitrite ions (NO_2_^−^) based on electrochemical changes or electrocatalytic sensing mechanisms. The roles of the Au-NPs in nitrite sensing include amplification of the sensing signal due to their high surface-area-to-volume ratio and excellent catalytic properties. These properties enhance the sensitivity of the nitrite sensor, allowing the detection of low concentrations of nitrite ions in samples. Moreover, SrTiO_3_ is a semiconductor material with a wide bandgap; its electronic properties can be modified by doping or surface functionalization, making it suitable for sensing applications. When exposed to nitrite ions, SrTiO_3_ undergoes changes in its electrical conductivity or surface potential, which can be used for sensing purposes. The surfaces can be engineered to exhibit high reactivity toward nitrite ions. Functionalization of the SrTiO_3_ surface with specific molecules or ions enhances its selectivity toward nitrite ions, enabling detection of nitrites in complex sample matrices. This plays a crucial role in nitrite sensing by providing a platform with tunable electronic properties, high reactivity toward nitrite ions, and compatibility with sensing devices, enabling the development of robust and sensitive nitrite sensors for various applications involving environmental monitoring, food safety, and biomedical diagnostics. The significant roles of Au-NPs in nitrite sensing also involve serving as transducers, signal amplifiers, and platforms for selective detection, thereby enabling the development of sensitive, selective, and reliable nitrite sensors for various applications involving environmental monitoring, food safety, and healthcare.

Herein, we report the development of Au-NPs/PPyC/SrTiO_3_ nanocomposites (NCs) using ultrasonic techniques and utilization of these nanostructures for electrochemical sensing of nitrites in a buffer of 7.0 pH. To date, numerous NCs of polypyrrole (PPy) have been used to develop electrochemical sensors, such as humidity (Hussain et al., 2021), serotonin (Song et al., 2019), NH_3_ (Kharat et al., 2007), and DNA (Booth et al., 2011) sensors. In addition, many reliable nitrite electrochemical sensors have been fabricated to detect gaseous nitrites by applying PPy films (Navale et al., 2014a), PPy-WO_3_ CNs (carbon nanostructure) (Mane et al., 2015), CSA (Camphor sulfonic acid)-PPy (Navale et al., 2014b), Ag-PPy NCs (Karmakar et al., 2017; Zhang et al., 2024), and PPy/N-Multiwall Carbon Nanotube (MWCNT) (Liu et al., 2019). To the best of our knowledge and a literature survey, there are no available studies on this topic; thus, the present study involves development of Au-NPs/PPyC/SrTiO_3_ NCs embedded with PEDOT:PSS/glassy carbon electrode (GCE) and utilized for the first time in an electrochemical approach.

## Experimental

### Materials and methods

To prepare the hybrid NC materials, a block copolymer with a molecular weight of 12,600 g/mol, F127 surfactant, titanium (IV) butoxide (TBOT), zinc acetate, polypyrrole-doped carbon (PPyC), HAuCl_4_·4H_2_O, methylene blue, hydrochloric acid, acetic acid, ethanol, and methanol were utilized. All chemicals were purchased from Sigma-Aldrich and used as received without further purification.

### Preparation of SrTiO_3_

To prepare SrTiO_3_, pluronic F127 (2.4 g) was first dissolved in ethanol (30.0 mL). A mixture of 3.5 mL of TBOT, 0.74 mL of 35% hydrochloric acid, and 2.3 mL of acetic acid was added to this solution in a conical flask and stirred constantly at room temperature for an hour. Subsequently, approximately 2.23 g of Sr(NO_3_)_2_ was added to the mixture to form a mesophase of CH_3_COOH-TBOT-F127 using Sr:Ti in the molar ratio of 1:1. This solution was stirred continuously for 60 min, and the ethanol was evaporated by drying the resulting solution at 40°C and 40% humidity for 24 h. Finally, the gel slurry was aged at 65°C and heated at the rate of 1°C per minute for 4 h at 900°C in a muffle furnace to obtain the mesoporous SrTiO_3_ material.

### Synthesis of the Au-NPs/PPyC/SrTiO_3_ NC

Next, the 10% PPyC/SrTiO_3_ NC was prepared by the ultrasonication method. SrTiO_3_ (1.0 g) and PPy (0.1 g) were ultrasonicated in 100.0 mL of deionized (DI) water for 15 min, filtered carefully, and then washed with ethanol and DI water. Over the next 24 h, the wet precipitate was dried at 65.0°C, resulting in the formation of the 10% PPyC/SrTiO_3_ NC. To create a ternary framework, 1% Au-NPs was added to the NC using the photoreduction strategy, as reported previously (Faisal et al., 2022; Li Q. et al., 2023; Wang S. et al., 2024). A solution of HAuCl_4_·4H_2_O (1% Au) was added dropwise to 0.5 g of the 10% PPy/SrTiO_3_ suspension in 100 mL of DI water, and the suspension was placed under a mercury lamp (2.0 mW/cm^2^) for 12 h with continuous stirring. The resulting mixture was centrifuged 3–4 times violently for appropriate separation of the desired material. Thereafter, the obtained material washed with ethanol and DI water before being dried at 65°C for 24 h to obtain the final product: Au-NPs/PPyC/SrTiO_3_ NC.

### Characterization of the Au-NPs/PPyC/SrTiO_3_ NC

The prepared NCs were thoroughly characterized for functional, morphological, structural, elemental, and surface area analyses using conventional testing methods. The structural and crystalline properties of the Au-NPs/PPyC/SrTiO_3_ NC were analyzed by various techniques. A PANalytical (X’port) diffractometer was used for the powder X-ray diffraction (XRD) analysis with Cu K_1/2_ (1 = 154.060, 2 = 154.439 p.m.) radiation. The NC was then analyzed by field-emission electron microscopy (FESEM; JEOL-6300F) and high-resolution transmission electron microscopy (HRTEM; JEOL JEM-2100F-UHR) at 200 kV attached to a 1k charge coupled device (CCD) camera and a Gatan energy filter (GIF, 2001) to determine the morphology and structure. The binding energy of the Au-NPs/PPyC/SrTiO_3_ NC was determined by X-ray photoelectron spectroscopy (XPS) using a VGESCALAB 200R P Thermo VG system equipped with a hemispherical electron analyzer and MgKα radiation (hν = 1253.6 eV). The Brunauer–Emmett–Teller (BET) surface area and pores size variation were analyzed at 77 K on a Quantachrome: NOVA 4200 analyzer with overnight degassing at 200.0°C. We then analyzed the Halsey-equation-based adsorption data using the Barrett–Joyner–Halenda (BJH) model and also determined the BET surface area. Additionally, diffuse reflectance spectra collected between 200 and 800 nm were used for the bandgap energy study employing a Shimadzu UV-Vis 3600 spectrophotometer; the results were collected for each freshly generated NC sample.

### Development of the glassy carbon working electrode

**FIGURE 1:**
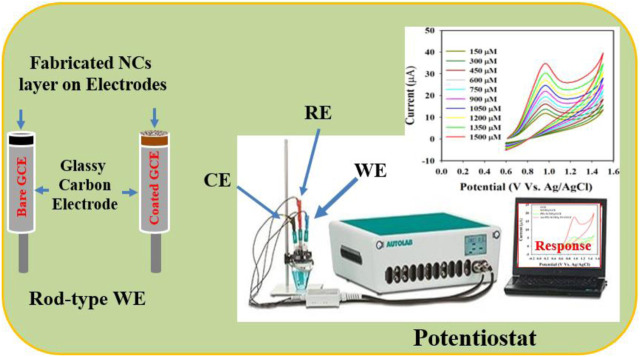
Schematic representation of the fabricated nitrite sensor probe with a modified glassy carbon electrode (GCE) and Auto-Lab assembly operation.

A GCE was modified using the Au-NPs/PPyC/SrTiO_3_ NC to create the working electrode (WE) of the sensor. This was achieved by preparing a NC-ethanol slurry, and the GCE’s flat surface was covered with a thin coating of the slurry before being air-dried in open air. About 10.0 µg of PEDOT:PSS (in a 5% ethanol solution) was added to maintain the strength of the GCE’s deposited layer. The modified GCE was then heated at 35°C for an hour. The desired electrochemical sensor was assembled using a potentiostat of Metrohm Autolab modules with the modified GCE as the WE, Ag/AgCl (saturated KCl) as the reference electrode, and Pt wire as the counter electrode, as shown in Figure 1. The electrochemical investigations were carried out via CV by diluting the nitrite in a phosphate buffer saline (PBS) medium at pH 7, with the concentrations ranging from 0.15 to 1.5 mM. The obtained data were then used to determine the sensor’s sensitivity, limit of detection (LOD), linear detection range (LDR), stability, and effect of pH during analysis of the fabricated material. The LOD was obtained from the equation LOD = 3.3σ/S, where σ and S refer to the standard deviation of the blank responses and slope of the calibration curve, respectively.

## Results and discussion

### FESEM and EDS analysis of Au‐NPs/PPyC/SrTiO_3_ nanocomposites

**FIGURE 2:**
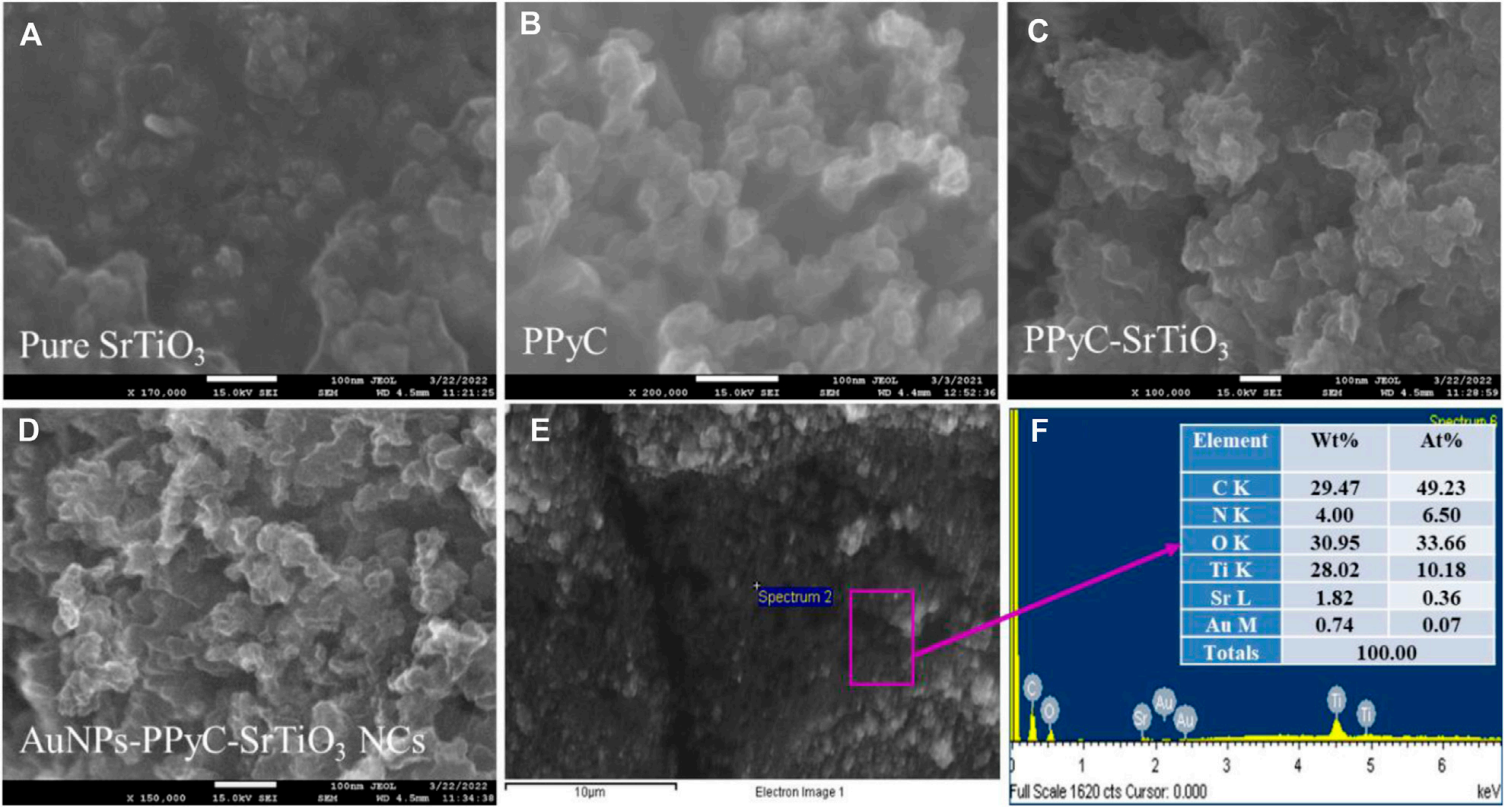
FESEM and EDS analyses of the nanocomposites (NCs). Magnified images of the **(A)** pure SrTiO_3_, **(B)** PPyC, **(C)**/PPyC/SrTiO_3_, and **(D)** Au-NPs/PPyC/SrTiO_3_ NCs; **(E)** EDS image of the Au-NPs/PPyC/SrTiO_3_ NC and **(F)** elemental composition of Au-NPs/PPyC/SrTiO_3_ NC.

The structural and morphological properties of the newly created Au-NPs/PPyC/SrTiO_3_ NCs and its constituent elements were analyzed by FESEM to obtain high-resolution images exhibiting the size, shape, and morphology. FESEM coupled with EDS was used to study the elemental composition of the NC. Figure 2 shows the FESEM and EDS images of the NC at high magnifications. As shown in Figure 2, the SrTiO_3_ particles exhibit irregular spherical shapes intercalated with PPy and Au-NPs. However, the prepared Au-NPs/PPyC/SrTiO_3_ NCs did not show any particular shapes or sizes at the nano level, as seen in the FESEM images in Figures 2A–C, and Figure 2D indicates that these are NCs. Similar observations can be made from Figure 2E for the elements. Elemental analysis of the Au-NPs/PPyC/SrTiO_3_ NCs using EDS revealed that they were composed of C (29.47%), O (30.95%), Ti (28.02%), Sr (1.82%), N (4.00%), and Au (0.74%), as seen in Figure 2F. Thus, EDS analysis confirmed the existence of Au, C, O, Ti, N, and Sr in the synthesized NCs.

**FIGURE 3:**
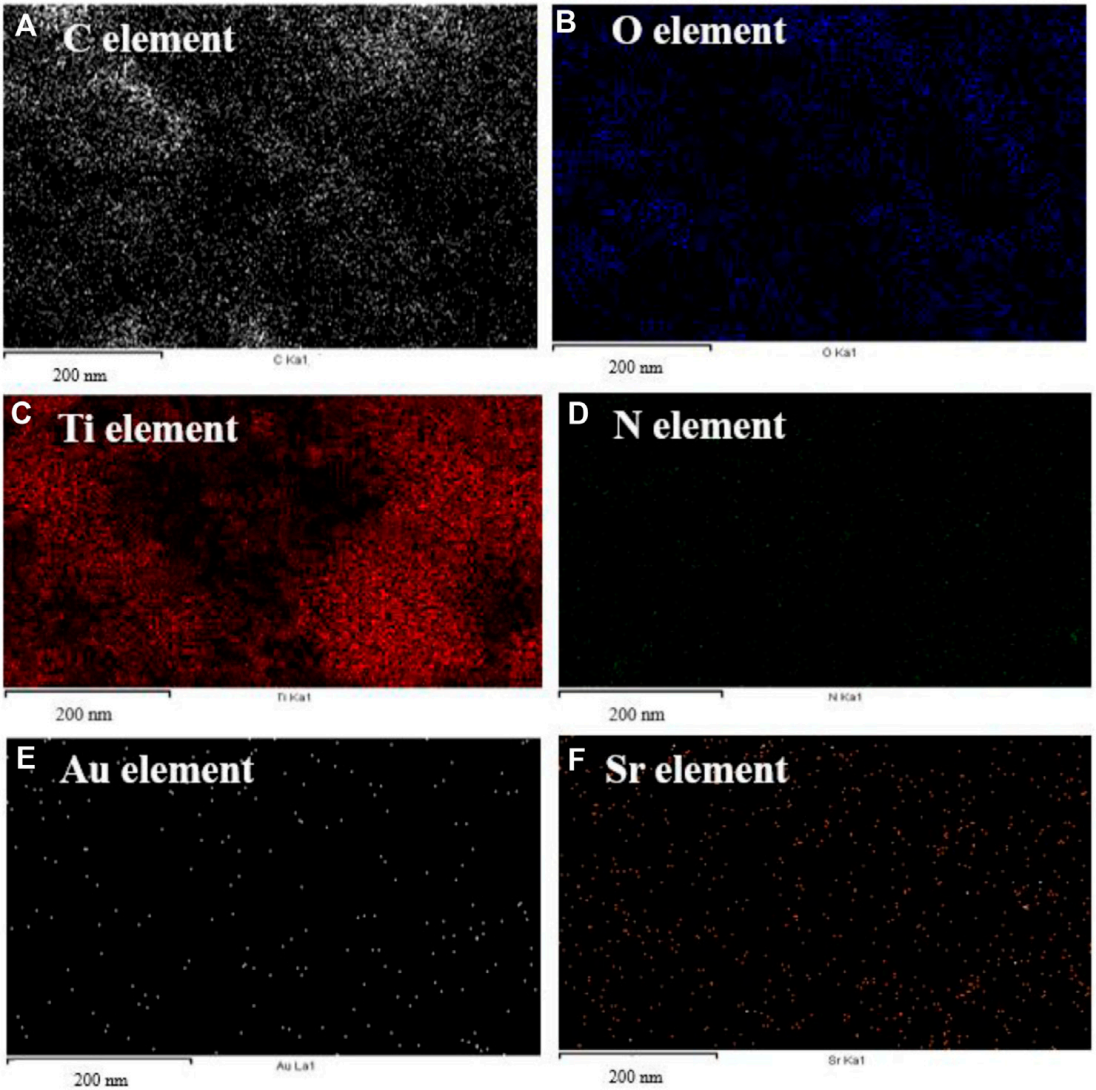
EDS mapping for elemental distribution in the Au-NPs/PPyC/SrTiO_3_ NC: **(A)** C, **(B)** O, **(C)** Ti, **(D)** N, **(E)** Au, and **(F)** Sr.

Elemental mapping of the Au-NPs/PPyC/SrTiO_3_ NCs involves spatially resolved analysis of the distribution of each element within the NC structure. This technique provides valuable insights into the composition, morphology, and distribution of the constituents of the Au-NPs/PPyC/SrTiO_3_ NC. The spatial distribution of each element in the NC is shown in Figure 3. Each pixel/color dot in the map represents a specific area of the target sample, and the intensity of the pixel/dot corresponds to the concentration of the element detected in the prepared Au-NPs/PPyC/SrTiO_3_ NC. Additionally, by analyzing the elemental mapping, we can identify regions rich in specific elements and assess the uniformity or segregation of different components within the Au-NPs/PPyC/SrTiO_3_ NC. Here, the elemental mapping of each element was obtained separately, as presented in Figure 3. These maps are analyzed to gain insights into the composition and distribution of the NC constituents, which are analyzed and presented for C in Figure 3A, O in Figure 3B, Ti in Figure 3C, N in Figure 3D, Au in Figure 3E, and Sr in Figure 3F. Thus, EDS mapping confirmed the existence and dispersion of Au, C, O, Ti, N, and Sr in the synthesized Au-NPs/PPyC/SrTiO_3_ NCs.

### HRTEM analysis of the Au-NPs/PPyC/SrTiO_3_ NC

**FIGURE 4:**
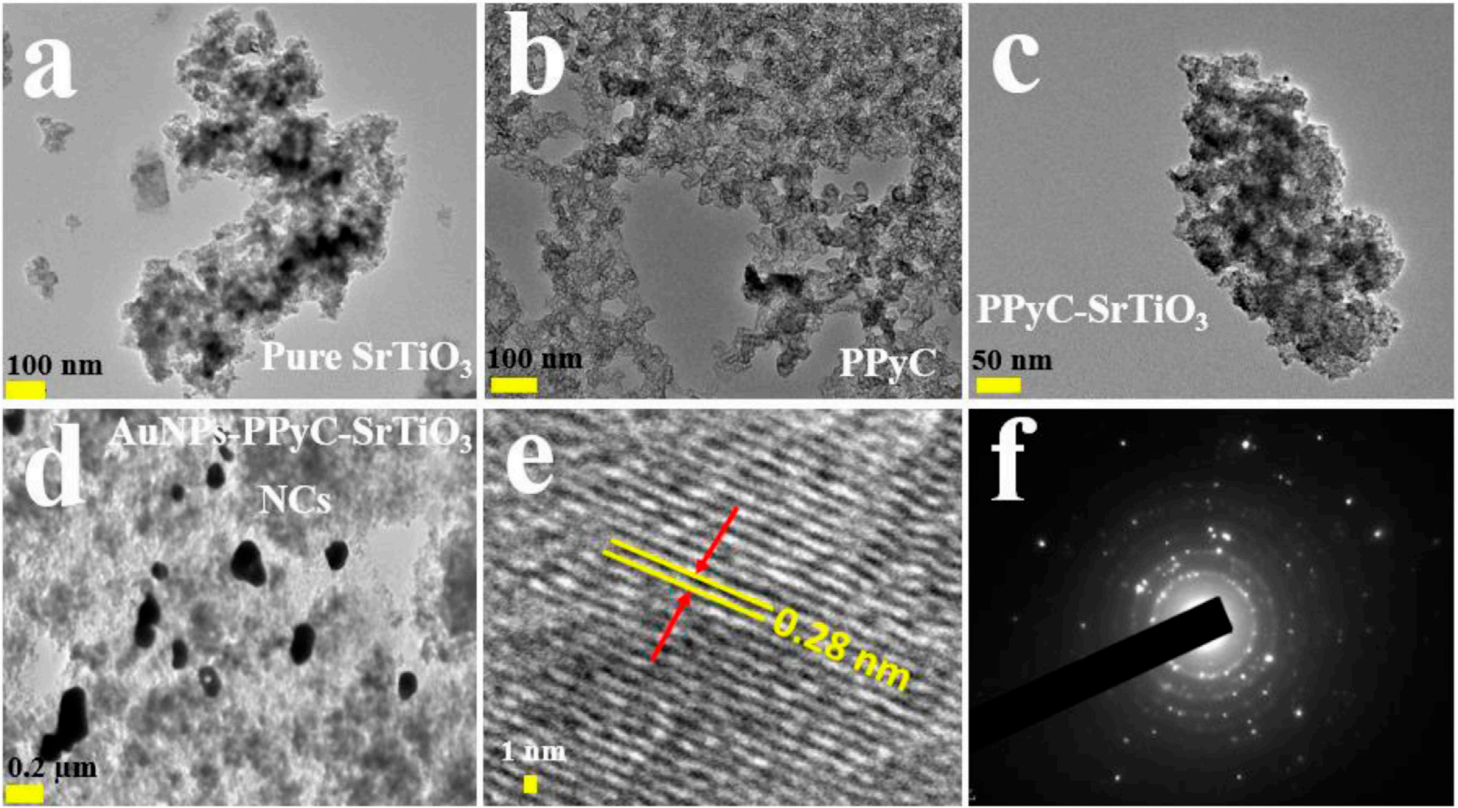
HRTEM analyses of the NCs. Magnified images of **(A)** pure SrTiO_3_, **(B)** PPyC, **(C)** PPyC/SrTiO_3_, and **(D)** Au-NPs/PPyC/SrTiO_3_ NCs; **(E)** lattice spacing and **(F)** selected-area electron diffraction pattern of the NC.

In this analysis, spherical-shaped nanoparticles ranging in size from 100.0 nm to 300.0 nm were found in the Au-NPs/PPyC/SrTiO_3_ NC, while the PPy polymer crystallized into a network of compact particle-shaped nanostructures. Figures 4A–C and Figure 4D demonstrate the findings of the HRTEM study, which validates the morphological structures of the Au-NPs, PPyC, and SrTiO_3_ separately. PPy is shown to be intercalated with SrTiO_3_ and Au-NPs in the HRTEM images. Figures 4E, F display the HRTEM and diffraction pattern results of the Au-NPs/PPyC/SrTiO_3_ NCs. Lattice fringes/spacing are visible in the HRTEM image of the Au-NPs/PPyC/SrTiO_3_ NC in Figure 4E, suggesting that the nanocrystal structure is well-ordered and devoid of displacements. Further evidence that the NC is crystalline was found when the d-spacing between lines in the lattice was determined to be 0.28 nm. In Figure 4F, we see the selected area electron diffraction (SAED) results of the probed Au-NPs/PPyC/SrTiO_3_ NC, which exhibits bright spots grouped in a concentric pattern, confirming the crystalline character of the ternary NC.

### XPS analysis of the Au-NPs/PPyC/SrTiO_3_ NC

**FIGURE 5:**
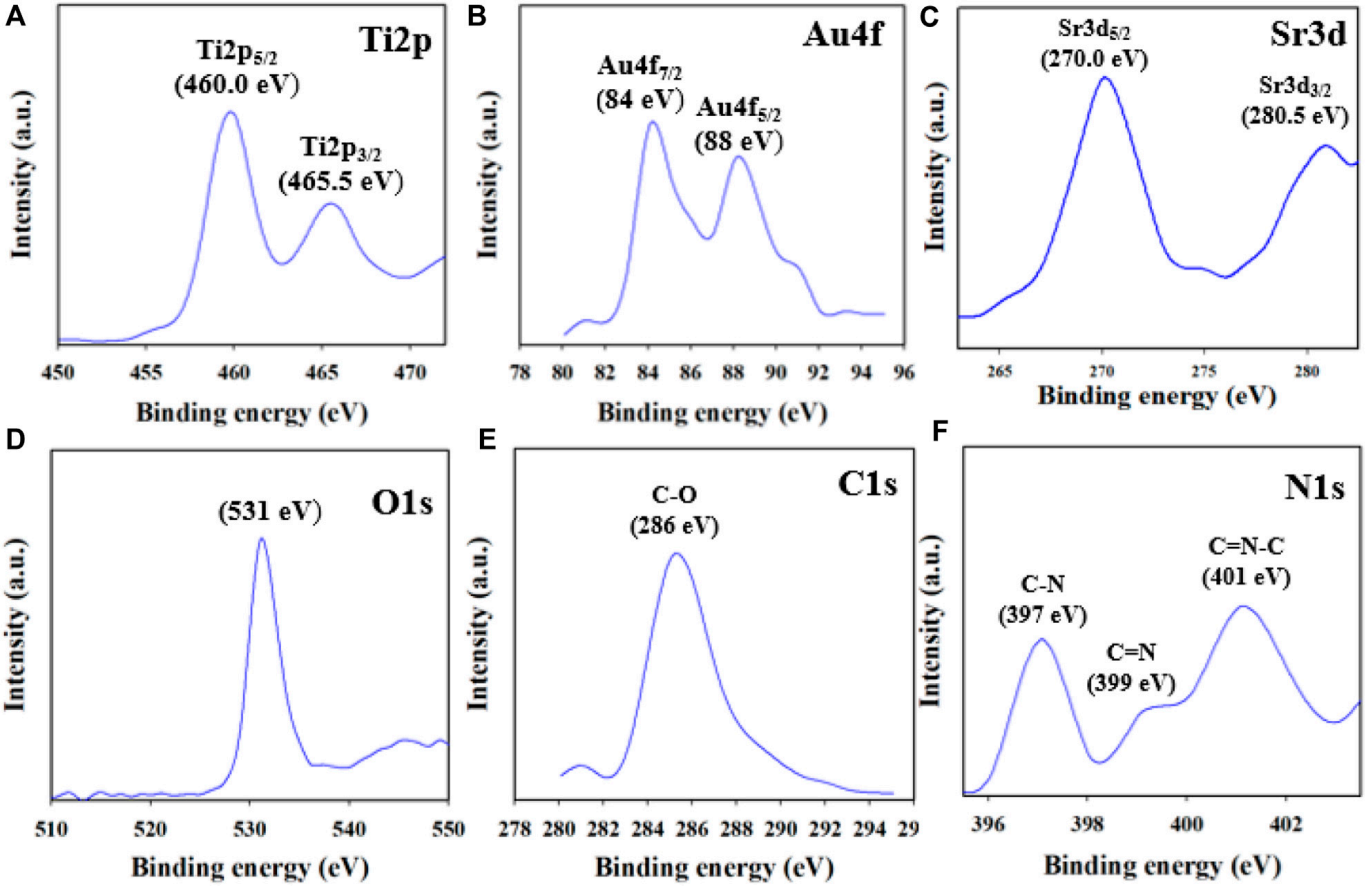
XPS results of the Au-NPs/PPyC/SrTiO_3_ NC: **(A)** Ti2p, **(B)** Au4f, **(C)** Sr3d, **(D)** O1s, **(E)** C1s, and **(F)** N1s.

Utilizing the XPS method, it is feasible to ascertain the oxidation states of the atoms in the NC. In this study, the XPS data of the synthesized Au-NPs/PPyC/SrTiO_3_ NCs were analyzed to gather valuable information regarding the chemical composition and oxidation states of the NC. As presented in Figure 5A, the Ti2p orbital was subdivided into two spin orbitals, Ti2p_3/2_ and Ti2p_1/2_, indicating Ti^4+^ ionization (Nawaz et al., 2019; Faisal et al., 2023b). The Au-NPs exhibited XPS peaks at binding energies of 84 and 88 eV for the 4f_7/2_ and 4f_5/2_ transitions, respectively, confirming the presence of Au-NPs in the NC, as shown in Figure 5B (Vitale et al., 2011; Chen et al., 2015). Figure 5C presents two peaks at 270 and 280.5 eV for Sr3d_5/2_ and Sr3d_3/2_, respectively, confirming the existence of Sr^2+^ in the prepared NCs (Atuchin et al., 2013; Li et al., 2017). The O1s orbital shown in Figure 5D displays a peak at 531 eV for Ti-O or Sr-O, confirming the oxidation state of Ti(II) (Bakhoum et al., 2022). The C1s XPS curve shown in Figure 5E represents the C-O bond (Xiao et al., 2017; Bourlier et al., 2018) while the N1s orbital in Figure 5F shows C=N and O-N peaks (Majumdar et al., 2012; Ravi et al., 2018). Overall, the XPS analysis confirmed the existence of Sr^+2^, Ti^4+^, Au-NPs, and O^2+^ as well as C-O, C=N, and O-N bonds in the synthesized NCs, providing valuable information regarding their chemical compositions and oxidation states.

### Crystallographic, optical absorbance, and surface area analyses

**FIGURE 6:**
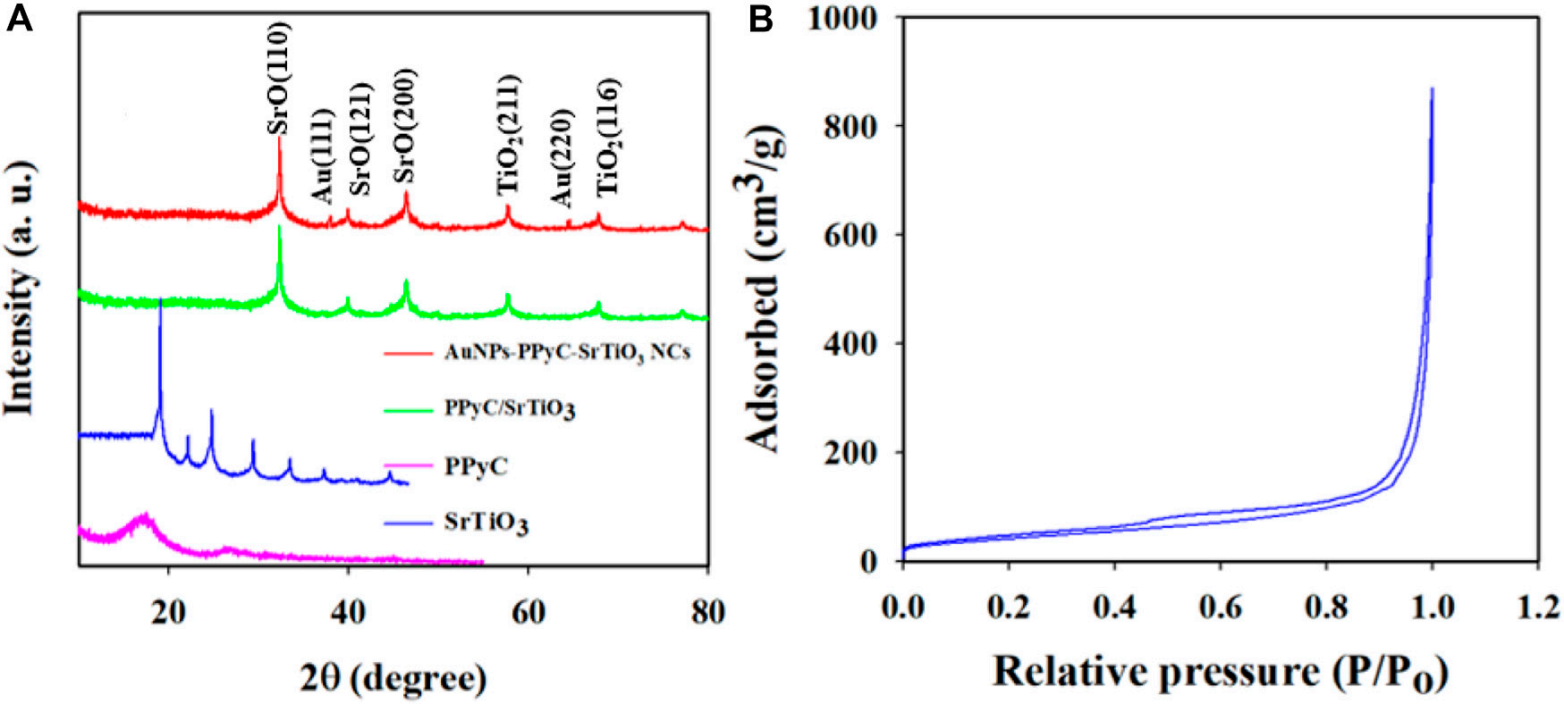
**(A)** Phase crystallinity analysis of the Au-NPs/PPyC/SrTiO_3_ NC with comparison of its constituent elements and **(B)** surface area of the NC by BET analysis.

The crystallographic study of the prepared NC was conducted via powder dispersion using X-rays, as shown in Figure 6A. The obtained data clearly indicate that the crystalline peaks of the synthesized Au-NPs/PPyC/SrTiO_3_ NC are SrO and TiO_2_ only. The crystallographic study revealed peaks for TiO_2_ corresponding to the (211) and (116) planes (Fang et al., 2012; Kaygili et al., 2017; Fu et al., 2023). Several crystalline peaks of SrO were also detected, corresponding to the (110), (121), and (200) planes, in accordance with a previous report (Tan et al., 2014; Tabah et al., 2017; Trang et al., 2021; Cai et al., 2023). Additionally, the XRD pattern for the prepared Au-NPs/PPyC/SrTiO_3_ NC showed two extra peaks at 38.12 and 64.38 eV for the (1 1 1) and (2 2 0) crystal planes compared to the PPyC/SrTiO_3_ sample, signifying the existence of Au NPs (JCPDS Card No. 01–1174). Finally, it is important to note that no contaminant peaks of any impurities were observed in the XRD investigations, confirming the formation of Au-NPs/PPyC/SrTiO_3_ NCs only (Faisal et al., 2023a; Zhao et al., 2024). The obtained XRD values suggest the development of a structure containing Au-NPs, SrO, and TiO_2_. The BET surface analysis was used to determine the active surface areas of the NCs using nitrogen adsorption and desorption, as demonstrated in Figure 6B. The calculated relative surface area of the Au-NPs/PPyC/SrTiO_3_ NC is 119.34 m^2^/g, indicating that the NC exhibits a good surface area favorable for electrocatalytic reactions. The pore size and pore volume (BJH results) of the Au-NPs/PPyC/SrTiO_3_ NCs were found to be 16.61 nm and 0.65 cm^3^/g, respectively.

### Electrochemical characterization of the Au-NPs/PPyC/SrTiO_3_ NC/GCE probe

**FIGURE 7:**
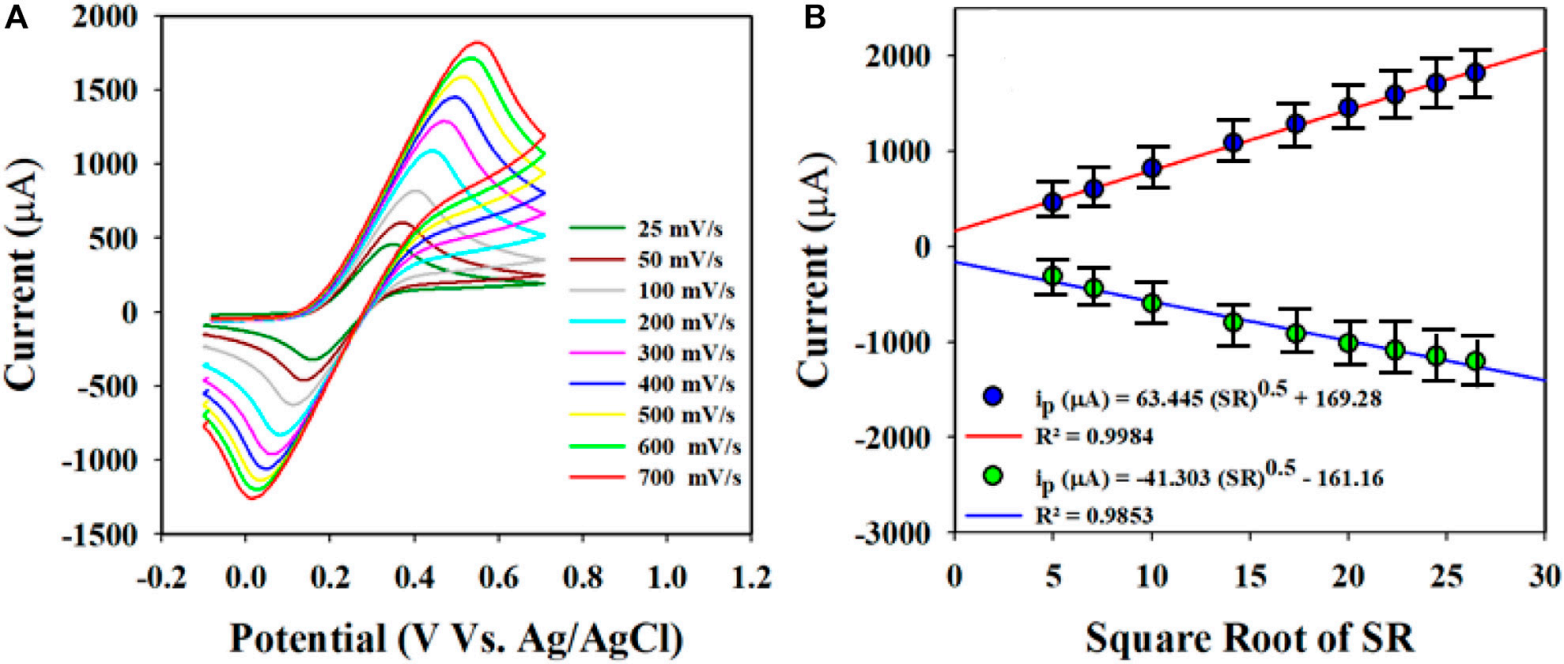
Electrochemical characterization of the Au-NPs/PPyC/SrTiO_3_ NC using cyclic voltammetry (CV): **(A)** investigation of the scan rate of the coated GCE using Au-NPs/PPyC/SrTiO_3_ NC in the oxidation reduction of 0.1 mM K_4_[Fe(CN)_6_]; **(B)** peak current *versus* square root of the SR for nitrite detection by CV with the Au-NPs/PPyC/SrTiO_3_ NC/GCE probe.

CV measurements were obtained at a scan rate of 25–700 mV/s to assess the molecular diffusional ability on the WE surface (Au-NPs/PPyC/SrTiO_3_ NC/GCE) using the PBS phase at pH 7.0 containing 0.1 mM K_4_ [Fe(CN)_6_], as presented in Figure 7A. Figure 7A displays a linear distribution of the peak currents measured during the oxidation/reduction of K_4_ [Fe(CN)_6_]. Therefore, good electrochemical performance of the modified electrode for detecting electroactive species is revealed in the current *versus* square root of the scan rate (SR) plot, as shown in Figure 7B. The equations (Equations 1, 2) representing the plot are also given.

$$i_{p}=63.445\;{\left(\text{SR}\right)}^{0.5}+169.28;\;R^{2}=0.9984\text{ at oxidation of }K_{4}\left[\text{Fe}{\left(\text{CN}\right)}_{6}\right]$$ (1)

$$\text{ip}=‐41.303{\left(\text{SR}\right)}^{0.5}\;‐161.16;\;R^{2}=0.9853\text{ at reduction of }K_{4}\left[\text{Fe}{\left(\text{CN}\right)}_{6}\right]$$ (2)

**FIGURE 8:**
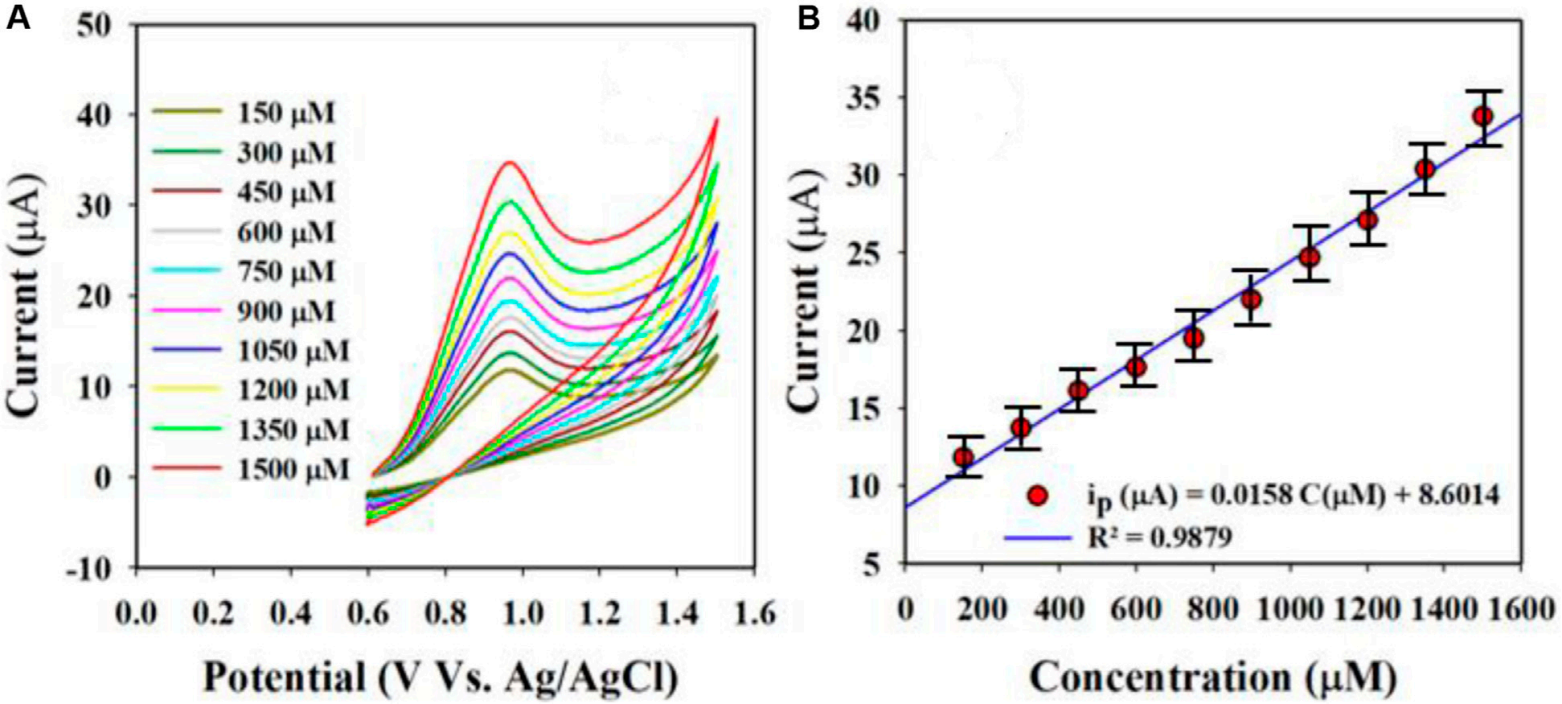
Nitrite is evaluated electrochemically through differential pulse voltammetry (DPV): **(A)** oxidation peak currents increased with concentration and **(B)** calibration curves of the nitrite sensor (current vs. concentration).

These equations indicate that the molecules are controlled by diffusion on the fabricated WE surface, as similar phenomena have been reported previously (Alam et al., 2022; Rahman et al., 2022; Faisal et al., 2023d; Li J. et al., 2023; Rahman et al., 2023). Nitrite was analyzed using the CV electrochemical method in a pH 7.0 conductive buffer solution, as presented in Figure 8A. The nitrite oxidation peak currents are shown to scale linearly with concentrations from 150.0 to 1500.0 µM. As shown in Figure 8B, the dispersed currents increase from 150.0 to 1500.0 µM, and this concentration range is determined to be the LDR for nitrite detection. The current vs. concentration plot is expressed by Equation 3 below:

$$i_{p}=0.0158\;C\left(µM\right)+8.6014;\;R^{2}=0.9879\text{ at Oxidation of Nitrite}$$ (3)

The built-in nitrite sensor’s sensitivity was determined using the slope of its calibration curve, and the best sensitivity for detecting nitrite was 0.5 µA/µM∙cm^2^. The LOD was determined to be 18.99 ± 0.95 µM for the nitrite sensor, and the LOD was calculated from the equation LOD = 3.3σ/S, where σ and S refer to the standard deviation of the blank responses and slope of the calibration curve, respectively.

Optimization of the Au-NPs/PPyC/SrTiO_3_ NC/GCE sensor probe

**FIGURE 9:**
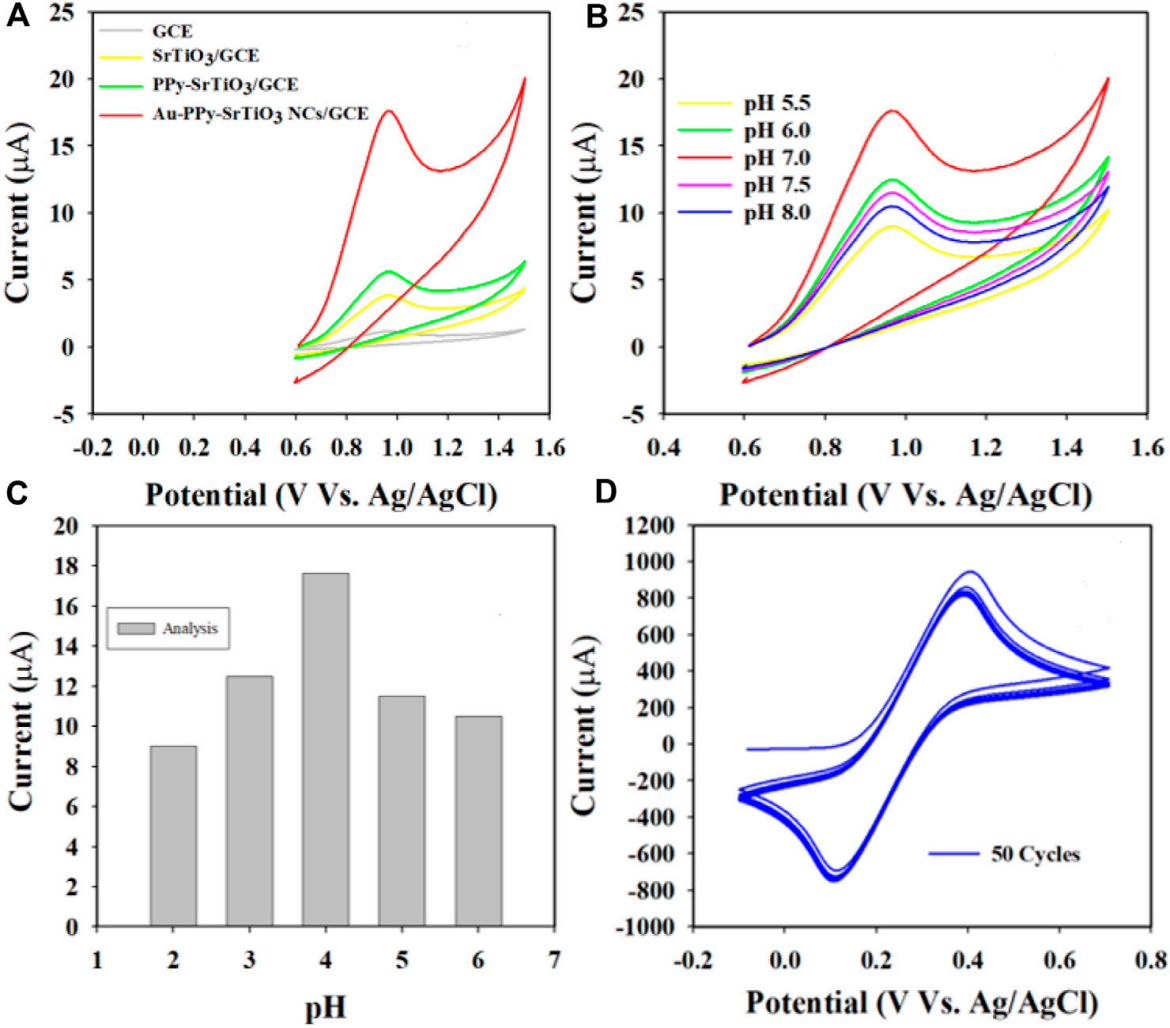
pH optimization in nitrite detection with the Au-NPs/PPyC/SrTiO_3_ NC/PEDOT:PSS fabricated GCE probe: **(A)** controlled experiment, **(B)** CV analysis of nitrite based on pH of the buffer, **(C)** bar diagram, and **(D)** stability performance of the working electrode.

In this approach, the WEs were fabricated using various compositions of the prepared target Au-NPs/PPyC/SrTiO_3_ NC, including the bare GCE, SrTiO_3_, and PPyC/SrTiO_3_, as presented in Figure 9. It is shown that the Au-NPs/PPyC/SrTiO_3_ NC/PEDOT:PSS/GCE exhibits the maximum oxidation current compared to the other constituent compositions in a pH 7.0 buffer containing 750 µM of nitrite using CV analysis. The pH of the buffer is a significant factor in the detection of nitrite compounds under identical conditions. Therefore, the oxidation performance of nitrite was also tested in a wide pH range (from acidic to basic; 5.5–8.0), as presented in Figures 9B, C. These results clearly demonstrate that efficient oxidation in the buffer containing 750.0 µM nitrite was obtained at pH 7.0 with the Au-NPs/PPyC/SrTiO_3_ NC/PEDOT:PSS/GCE probe through the CV technique, indicating that the chemical sensor probe has good selectivity and sensitivity for nitrite detection at physiological pH values.

Stable performance of the WE in an electrochemical reaction is a key characteristic for measuring reliability. The glassy carbon WE coated with Au-NPs/PPyC/SrTiO_3_ NC was examined in the analysis of 0.1 mM K_4_ [Fe(CN)_6_], as shown in Figure 9D. As presented, the 50 cycles of CV were almost indistinguishable, meaning that the working electrode was active, which indicates the high stability of the WE prepared with Au-NPs/PPyC/SrTiO_3_ NCs; this probe is expected to exhibit similar performances in other analytes.

**FIGURE 10:**
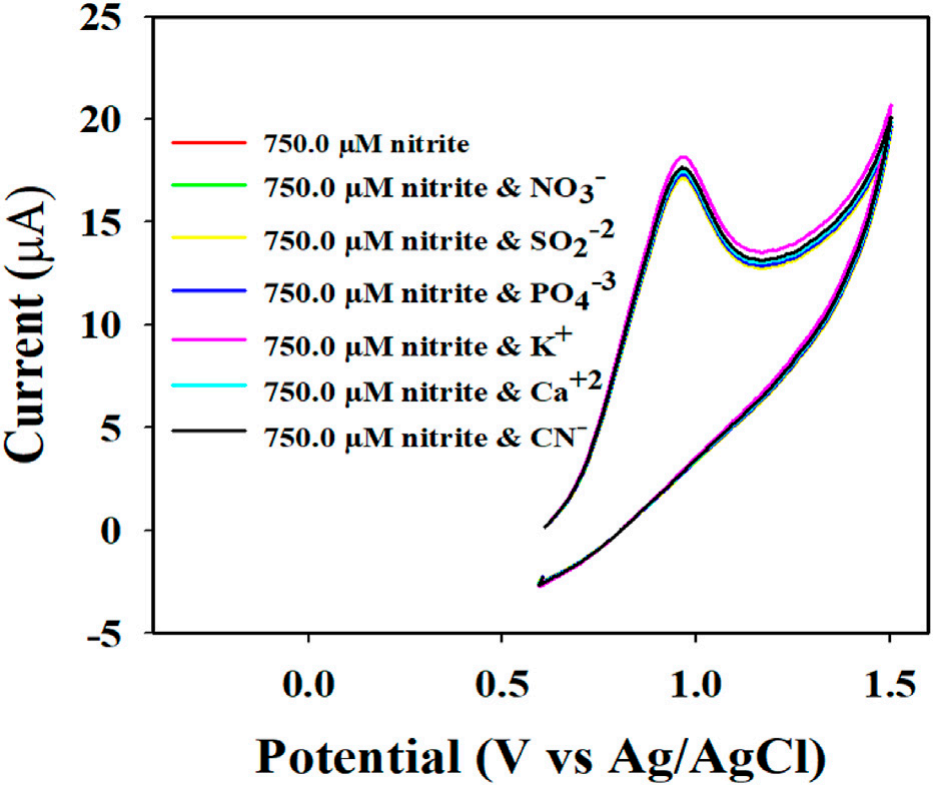
Electrochemical investigation of 750.0 µM nitrite in the presence of other analytes by the CV technique.

To investigate the nitrite electrochemical sensor based on Au-NPs/PPyC/SrTiO_3_ NC/PEDOT:PSS/GCE, interference was performed with a 750.0 µM nitrite solution in the presence of other ions, whose results are presented in Figure 10. The results are further analyzed and explored in Figure 10, where it is clearly illustrated that the nitrite electrochemical sensor did not show any interference in the presence of other ions such as NO_3_^−^, SO_2_^−2^, PO_4_^−3^, K^+^, Ca^+2^, and CN^−^. Thus, the Au-NPs/PPyC/SrTiO_3_ NC/PEDOT:PSS/GCE electrochemical sensor probe is selective toward only nitrites under identical conditions.

To validate the results of this electrochemical sensor study, comparisons were performed with similar studies using different sensing substrates, as shown in Table 1 (Zhang et al., 2013; Ma et al., 2014; Singh et al., 2019; Asiri et al., 2020; Rashed et al., 2020; Manikandan et al., 2021; Faisal et al., 2023c). From Table 1, it is seen that the Au-NPs/PPyC/SrTiO_3_ NC/PEDOT:PSS/GCE sensor provides better results than other modified electrodes, with reliable parameters in terms of the sensitivity, LDR, and LOD.

**TABLE 1:** Comparative analysis of the nitrite sensor probe based on different sensing substrates via the electrochemical technique.

Electrode materials	LOD	LDR	Sensitivity	Ref.
*AuNPs/GO*	6.00 nM	0.02∼0.153 μM	---	Singh et al. (2019)
*Au-G-PANI/GCE*	0.01 μM	0.1∼200 μM	---	Ma et al. (2014)
*Au/Co*_*3*_ *O*_*4*_ */GCE*	0.11 μM	1.0∼4,000.0 μM	---	Manikandan et al. (2021)
*Cu-NDs/RGO/GCE*	0.4 μM	1.25 µM to 13 mM	2.14 μA/mM^‐1^cm^2^	Zhang et al. (2013)
*rGO/ZnO/GCE*	1.18 μM	20∼520 µM	0.32 μA/µM^‐1^cm^2^	Rashed et al. (2020)
*GS/MWCNT/CD*	1.65 μM	5 μM to 6.75 mM	---	Zhang et al. (2011)
*Au@PPy-C/C_3_N_4_ NCs/GCE*	1.11 ± 0.05 µM	1.5∼22.5 µM	91.2 µA/µM^‐1^cm^2^	Faisal et al. (2023c)
*La_2_CuO_4_/Naf/GCE*	0.04 µM	0.1–480 µM	12.0 µA/µM^‐1^cm^2^	Asiri et al. (2020)
*Au-NPs/PPyC/SrTiO*_*3*_ *NCs/PEDOT:PSS/GCE*	18.99 µM	150∼1,500 µM	500.0 nA/μM^‐1^cm^2^	*This study*

### Detection of nitrites with the Au-NPs/PPyC/SrTiO_3_ NC/GCE probe

**SCHEME 1:**
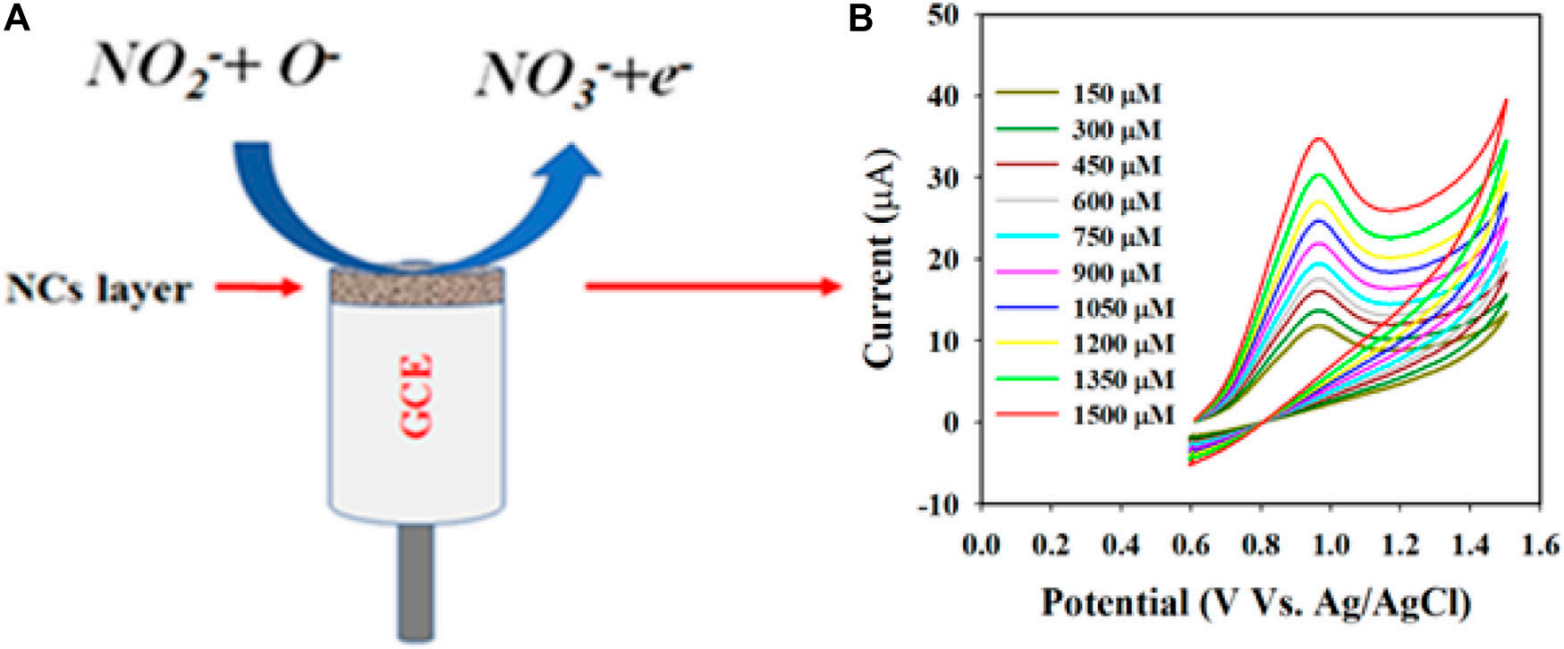
Schematic representation of the detection mechanism of nitrite with the Au-NPs/PPyC/SrTiO_3_ NC/PEDOT:PSS fabricated GCE: **(A)** electrochemical oxidation of nitrite (NO_2_^−^) on the fabricated electrode and **(B)** conversion of NO_2_^−^ to NO_3_^−^ depending on the injected concentration in the electrochemical cell.

The detection mechanism for nitrite (NO_2_^−^) is illustrated in Scheme 1. Initially, H_2_O molecules are adsorbed onto the Au-NPs/PPyC/SrTiO_3_ NC/PEDOT:PSS/GCE probe surface, leading to electrocatalytic oxidation reaction and formation of H^+^ and O^−^ ions. In the second stage, the NO_2_^−^ ions react with O^−^ ions and are oxidized to nitrate (NO_3_^−^) ions, generating free electrons on the Au-NPs/PPyC/SrTiO_3_ NC surface. This process leads to a significant increase in the conductivity of the fabricated electrode in the PBS phase, as shown in the reactions of Schemes 1A, B. The oxidation of nitrite to nitrate along with the production of electrons is a typical electrochemical reaction, and similar electrochemical oxidation reactions of nitrite (NO_2_^−^) have been reported elsewhere (Asiri et al., 2020; Rashed et al., 2020; Faisal et al., 2023c; Suiyi et al., 2024). The proposed detection mechanism highlights the efficient catalytic activity of the prepared Au-NPs/PPyC/SrTiO_3_ NC/GCE probe toward nitrite detection, providing valuable insights into the electrochemical processes involved (Equations 4–6) in the detection of nitrite using the MC (Zhang et al., 2013; Zhu et al., 2017; Fu et al., 2020; Rashed et al., 2020).

$$\text{NaN}O_{2}\;\to \;Na^{+}+NO_{2}^{‐}$$ (4)

$$H_{2}O\;\to \;2H^{+}+O^{‐}$$ (5)

$$NO_{2}^{‐}+O^{‐}\;\to \;NO_{3}^{‐}+e^{‐}$$ (6)

Real sample analysis the with Au-NPs/PPyC/SrTiO_3_ NC/GCE probe

In the final step to validate the sensor for the proposed applications, the prepared Au-NPs/PPyC/SrTiO_3_ NC/PEDOT:PSS/GCE probe was used to measure various real samples collected from the environment, as listed in Table 2. The electrochemical analysis involved using the recovery technique and an electrochemical approach. Figure 8B shows a calibration curve for the desired nitrite concentration. The obtained results are presented in Table 2, indicating successful detection of nitrites in different real samples from the environment using the Au-NPs/PPyC/SrTiO_3_ NC/PEDOT:PSS/GCE probe and electrochemical method. The sensor was found to be reliable and satisfactory, which indicates its potential for various environmental monitoring applications. Thus, the fabricated sensor with Au-NPs/PPyC/SrTiO_3_ NC/PEDOT:PSS/GCE can be implemented in microsized electrochemical electrodes/devices for *in situ* usage to allow continuous measurement of nitrite ions in various environmental samples via the electrochemical approach.

**TABLE 2:** Recovery approach applied to the analyses of real samples using a sensor probe made of Au-NPs/PPyC/SrTiO_3_ NC/PEDOT:PSS/GCE.

Real sample	Added nitrite conc. (µM)	Measured nitrite Conc.^a^ by the Au-NPs/PPyC/SrTiO_3_ NC (µM)			Average recovery^b^ (%)	RSD^c^ (%) (n = 3)
		R1	R2	R3		
Underground water	750	742	745	743	99.11	0.205
Sea water	750	755	748	753	100.26	0.479
Tap water	750	739	740	755	99.20	1.204

^a^ Calculations using the Au-NPs/PPyC/SrTiO_3_ NC/GCE were averaged across three repetitions (signal-to-noise ratio = 3).

^b^ Measurement or calculation of nitrite concentration. (Unit: µM).

^c^ Accuracy from three replicated measurements (R_1_, R_2_, and R_3_) is represented by the relative standard deviation (RSD) value.

## Conclusion

In this study, Au-NPs-conjugated PPyC/SrTiO_3_ NCs were deposited on GCEs to develop efficient nitrite electrochemical sensors with more conductive sensor-substrate using PEDOT:PSS as a conducting binder. The Au-NPs/PPyC/SrTiO_3_ NCs were initially synthesized through ultrasonication, followed by photoreduction, resulting in a morphology that promotes electrochemical reactions for detecting nitrites under ambient conditions via the three-electrode system. The Au-NPs/PPyC/SrTiO_3_ NC/PEDOT:PSS/GCE sensor probe exhibits high sensitivity, high LOD, and large LDR, indicating its potential for various environmental monitoring applications. To validate its practical application, the Au-NPs/PPyC/SrTiO_3_ NC/PEDOT:PSS/GCE probe was directly tested on real environmental samples, and satisfactory performance was observed with the electrochemical technique through the recovery approach. This electrochemical nitrite sensor probe, which uses a novel NC material, provides an easy-to-use tool for detecting nitrite levels and has significant potential for environmental and healthcare applications. The present study therefore provides valuable insights into the development of efficient and reliable sensors for environmental monitoring, facilitating real-time detection and quantification of nitrite levels in various environmental samples.

## Data Availability

The original contributions presented in the study are included in the article/Supplementary Material, and any further inquiries may be directed to the corresponding authors.
